# Enzyme-free release of adhered cells from standard culture dishes using intermittent ultrasonic traveling waves

**DOI:** 10.1038/s42003-019-0638-5

**Published:** 2019-10-29

**Authors:** Yuta Kurashina, Chikahiro Imashiro, Makoto Hirano, Taiki Kuribara, Kiichiro Totani, Kiyoshi Ohnuma, James Friend, Kenjiro Takemura

**Affiliations:** 1 Department of Mechanical Engineering, Faculty of Science and Technology, Keio University, 3-14-1 Hiyoshi, Kohoku-ku, Yokohama 223-8522 Japan; 2 Department of Materials Science and Engineering, School of Materials and Chemical Technology, Tokyo Institute of Technology, 4259 Nagatsutacho, Midori-ku, Yokohama, 226-8503 Japan; 3 School of Science for Open and Environmental Systems, Graduate School of Science and Technology, Keio University, 3-14-1 Hiyoshi, Kohoku-ku, Yokohama 223-8522 Japan; 4 Department of Materials and Life Science, Faculty of Science and Technology, Seikei University, 3-3-1 Kichijoji Kitamachi, Musashino, Tokyo 180-8633 Japan; 5 Department of Pharmacy, Yasuda Women’s University, 6-13-1 Yasuhigashi, Asaminami-ku, Hiroshima, 731-0153 Japan; 6 Department of Science of Technology Innovation, Nagaoka University of Technology, 1603-1 Kamitomioka-cho, Nagaoka, Niigata 940-2188 Japan; 7 Department of Bioengineering, Nagaoka University of Technology, 1603-1 Kamitomioka-cho, Nagaoka, Niigata 940-2188 Japan; 8 Center for Medical Devices and Instrumentation, Department of Mechanical and Aerospace Engineering, University of California, San Diego, CA 92093 USA

**Keywords:** Assay systems, Tissue engineering

## Abstract

Cell detachment is essential in culturing adherent cells. Trypsinization is the most popular detachment technique, even though it reduces viability due to the damage to the membrane and extracellular matrix. Avoiding such damage would improve cell culture efficiency. Here we propose an enzyme-free cell detachment method that employs the acoustic pressure, sloshing in serum-free medium from intermittent traveling wave. This method detaches 96.2% of the cells, and increases its transfer yield to 130% of conventional methods for 48 h, compared to the number of cells detached by trypsinization. We show the elimination of trypsinization reduces cell damage, improving the survival of the detached cells. Acoustic pressure applied to the cells and media sloshing from the intermittent traveling wave were identified as the most important factors leading to cell detachment. This proposed method will improve biopharmaceutical production by expediting the amplification of tissue-cultured cells through a more efficient transfer process.

## Abstract (web summary)

Yuta Kurashina et al. present an enzyme-free cell detachment method that employs the acoustic pressure and sloshing in medium from intermittent traveling waves. This proposed method will improve biopharmaceutical production by expediting the amplification of tissue-cultured cells through a more efficient transfer process.

## Introduction

Cell culturing underpins many biotechnological applications, including the production of biopharmaceuticals, biological proteins, tissue engineering, and gene transfection. The development of ~70% of all biopharmaceuticals currently involves mammalian cell culture procedures^1^. The number of biopharmaceuticals in the drug pipeline is rapidly increasing and the demand for biologics is expected to likewise increase over the next several decades^2^. Furthermore, research on stem cells, such as induced pluripotent stem cells^3^, has led to their clinical application in tissue engineering over the past several years^4^. These efforts highlight a growing need to efficiently culture and provide mass quantities of these cells.

There are two cell culture methods for efficient growth culture: monolayer and suspension. Traditionally, cells have been cultured in monolayers^5^, with the cells adherent on flat surfaces and requiring detachment with enzymes to release the cells before redeposition and adhesion: cell passage. Suspension culture methods have also been used in research, though careful monitoring is required to control, for example, the size of spheroids^6^ generated with a suspension culture. Adhesion agglomeration due to contact inhibition^7^ and maintenance of appropriate levels of oxygen, metabolites, and signaling molecules^8^ are required. Further, the environment each cell experiences in suspension cultures is different. Those cells on the outside of spheroids experience fluid shear from agitation, rapid chemical concentration changes, and ample nutrient inflow and waste extraction flow, all absent for cells on the interior of the spheroids. These differences lead to heterogeneity^9^ and a necrotic core, certainly desirable for representing actual tissue behavior in experiments, but a problem in mass culturing cells through deterioration of the overall quality of the cell culture.

As a consequence, and due to its handling ease, monolayer cell culturing has remained the predominant method for decades, requiring seeding, culture, detachment, and collection of the cells in a culture dish or flask^10^. Despite its predominance, there are important drawbacks to the method with but few improvements over the years, notably in cell passage. Protease is an enzyme that cuts off peptide bonds in proteins and is responsible for cell surface damage when cells are detached from a culture dish or flask^11^. Trypsin is one of the most important proteases, as it is widely used in culturing to detach the cells, and therefore is problematic due to the damage it causes to cell membranes^12–15^. A flow cytometer may be used to detect such damage through a decrease of cellular proteins, which has been shown to be dependent upon the cells’ time of immersion in trypsin solutions^16^. Prolonged trypsin treatment delays the first cell division and can adversely impact the proliferation of adherent cells^17^. The trypsinized cells may recover most of their surface proteins, they typically require 8–24 h to do so, but some of the expressed proteins after trypsinization were not reversible^18^. Hence the enzyme-free cell detachment method is better for continued cell culturing. Automated cell culture systems, which show improved culture efficiency, have manipulators to handle cell culture dishes and flasks and to inject or suction solutions. These automated systems help improve the efficiencies of the seeding, culturing, and collection processes^18^, though they still employ trypsin alongside robotic shaking and pipetting with robotics^19^, still damaging the cells. An enzyme-free cell detachment method, if possible, may offer substantial benefit in improving cell culture performance and quality.

One potential approach for cell detachment employs temperature-responsive polymers on cell culture surfaces^20^. When the temperature is lowered, these surfaces are rapidly hydrated, become hydrophilic, and cause spontaneous cell release. This method has been used to generate cell sheets^21^ and tissues^22^. However, the major drawback of this method is the necessity of the temperature-responsive polymer on the culture surface that is both expensive and prone to accidental or premature cell release. Further, this method requires skilled technicians trained to use it, adding to the cost. Finally, because these materials require substantial treatment to enhance cell adhesion and precision in handling, they have so far not been used in automated cell culture systems. The problem of effective and damage-free cell detachment remains.

Here we show an enzyme-free cell detachment method using acoustic pressure and sloshing caused by intermittent ultrasonic traveling waves formed from an ultrasonic transducer in a clinically ubiquitous cell culture dish. This approach provides a method for cell detachment with no impact on cellular viability and therefore can be used for efficient cell culturing in biotechnology applications.

## Results

### Design and fabrication of the cell detachment system

**Fig. 1:**
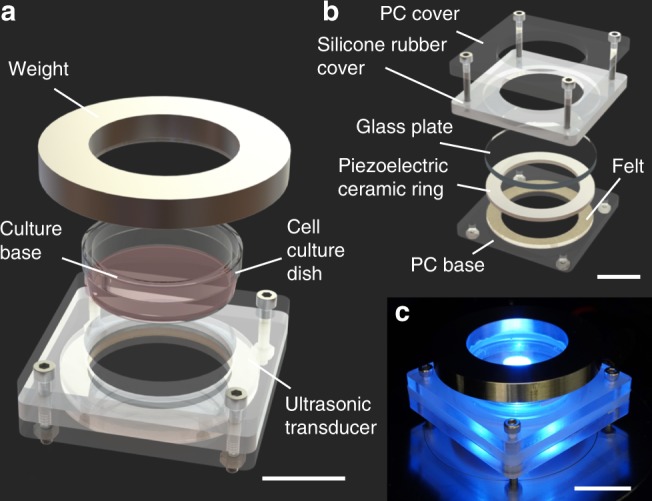
The cell detachment device using ultrasonic vibration. The cell detachment system, showing **a** how the culture dish is mounted in it and **b** its components. **c** The system is convenient for fluorescence microscopy, for example. All scale bars represent 20 mm

The cell detachment system, shown in Fig. 1a, is composed of an ultrasonic transducer, a glass plate and a piezoelectric ceramic ring to hold a cell culture dish. Figure 1b shows the ultrasonic transducer’s components. Ultrasound is passed to cells present in the dish via glycerol, which serves as an acoustic couplant that bridges the transducer-dish gap. The configuration of the system and dimensions are provided in the experimental setup in the Methods section, and Fig. 1c, Supplementary Fig. 1, and Supplementary Table 1. The details of the vibration conditions used in this study are described in the Measurement of vibration characteristics subsection in Methods.

### Evaluation of cell detachment

We next evaluated the cell detachment capability of the proposed method against conventional trypsinization as a control. As detailed in the Methods, Chinese hamster ovary (CHO) cells were seeded onto the cell culture dish and cultured in serum-supplemented medium (SSM), which is widely used for CHO cell culture, for a period of incubation time. This was followed by culturing in serum-free medium with Insulin-Transferrin-Selenium supplement (SFM) for an additional incubation time. The total incubation time was held constant at 48 h. The SFM does not contain adherence proteins, and thus the SFM does not prevent cell detachment through the action of these proteins during acoustically driven cell release. After switching the media from SSM to SFM, the ultrasound was activated, or, for the control, trypsinization was used.

**Fig. 2:**
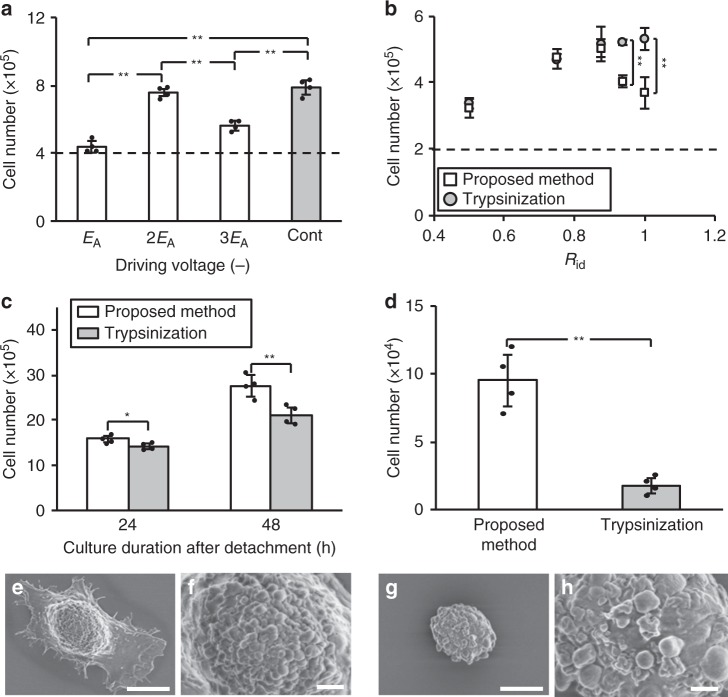
Determination of the appropriate voltage and SSM/SFM incubation conditions, and evaluation of the proliferation and adhesion ability. **a** Evaluation of the proposed method for cell detachment while varying the ultrasound appropriate excitation voltage. Cells (4 × 10^5^) were cultured with serum-supplemented medium (SSM) for 24 h. The SSM was replaced by serum-free medium with Insulin-Transferrin-Selenium supplement (SFM) and cells were subsequently cultured for 24 h (*R*_id_: 0.5). The cells were then either detached by our device (frequency: 29–31 kHz, sweep cycle: 10 ms, exposure duration: 5 min) with a driving voltage as indicated (*E*_A_, 2*E*_A_ or 3*E*_A_) or trypsinization (exposure duration: 5 min). Note that *E*_A_ = 100 V, the total cell cultured duration of all conditions was 48 h (SSM and SF SFM), and the immersion duration ratios (*R*_id_) were calculated using *R*_id_ = (time while exposed to SSM)/(time while exposed to SSM and SFM). **b** The number of detached cells was determined as a function of the exposure time ratio *R*_id_. The cells (2 × 10^5^) were seeded for culture with *R*_id_ ∈ {0.5, 0.75, 0.875, 0.9375, and 1}, then detached either with our method (frequency: 29–31 kHz, swept speed: 50 Hz, exposure duration: 5 min, driving voltage: 2*E*_A_) or trypsinization (exposure duration: 5 min). Data are shown as mean ± SD; *n* = 4, **p < 0.01, all *p*_SW_ > 0.05. **c** Comparison of the **c**ell proliferation detached with the proposed method (*R*_id_: 0.875; frequency: 29–31 kHz, upward and downward speed: 50 Hz, exposure duration: 5 min, driving voltage: 2*E*_A_) versus trypsinization (exposure duration: 5 min). Detached cells were cultured for 24 and 48 h. **d** The graph shows the number of adherent cells on the cell culture dish at 5 min after detachment for evaluation of adhesion ability. **e**–**h** Scanning electron microscopy images of (**e**, **f**) cells detach**e**d by the proposed method using ultrasonic vibration and (**g**, **h**) cells detached by trypsinization (mean ± SD, *n* = 4, *p < 0.05, **p < 0.01, all *p*_SW_ > 0.05). Scale bars, 5 µm (**e**, **g**); 1 µm (**c**, **d**)

We first determined the appropriate input voltage by evaluating the number of cells detached compared to trypsinization. Cells were first cultured in the SSM for 24 h, followed by the SFM for 24 h. We define an immersion duration ratio from the times the cells spend in the two media, *R*_id_ = (time while exposed to SSM)/(time while exposed to SSM and SFM), such that *R*_id_ = 0.5 here. Compared with trypsinization, the method detached only 55.6% as many cells with a voltage of *E*_A_ = 100 V (Fig. 2a). Doubling the voltage to 2*E*_A_ increased the number of detached cells to 96.2%, and because it was notably superior to the other choices, we selected 2*E*_A_ for all subsequent experiments. Details of *E*_A_ and *R*_id_ are described in Methods.

We next evaluated the number of detached cells using our method (input voltage: 2*E*_A_) for several SSM/SFM incubation conditions (*R*_id_: 0.5, 0.75, 0.875, 0.9375, and 1) (Fig. 2b). We observed the highest number of detached cells when *R*_id_ was 0.875, corresponding to an immersion duration in SSM medium for 42 h and afterwards in an SFM for 6 h, with no significant difference observed between the cells detached by proposed method and by trypsinization. From these results, we selected the following parameters for further study: *R*_id_: 0.875, range of input signal frequency: 29–31 kHz, input voltage: 2*E*_A_, modulation period for sine frequency-modulated input signal over 29 to 31 kHz: 0.02 s, and exposure duration: 5 min.

### Cell proliferation and initial adhesion of cells

To evaluate the viability of the cells detached by the proposed method versus trypsinization, we reseeded the detached cells, cultured the cells for several days, and then quantified the number of cells. The number of detached cells was significantly greater using the proposed method compared with trypsinization, as shown in Fig. 2c which compares the two methods after 24 and 48 h incubation. After 24 h, the proposed method produced a modest 11.2% improvement (*p* = 0.027, Ryan’s method) that grows to a remarkable 31.2% at 48 h (*p* = 0.009, Ryan’s method); these differences were significant.

We then evaluated the cells’ post-detachment adhesion to a new surface after reseeding, as shown in Fig. 2d as a comparison between the two detachment methods. The number of adhered cells after 5 min was significantly (5.4 times) greater using the proposed method (*p* = 0.001, Ryan’s method). To determine why this was the case, scanning electron microscopy was used to image the cell morphology post-adhesion, as shown in Fig. 2e–h. The cells detached by the proposed method show rough outer surfaces with observable pseudopodia (Fig. 2e, f). By contrast, the cells detached by trypsinization show smooth outer surfaces (Fig. 2g, h), because trypsinization has partially digested the surface proteins and the extracellular matrices^23^. These results suggest that the pseudopodia of the cells detached by the proposed method suffer less damage than cells detached by trypsinization. All data reported in these results—and in fact in the results to come—were confirmed to have normal distributions based upon the standard Shapiro–Wilk test.

### Surface proteins of detached cells

**Fig. 3:**
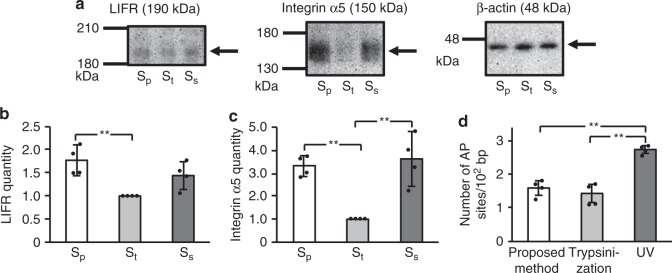
Evaluation of cell surface proteins and DNA damage in CHO cells post-detachment. **a**–**c** Detached cells, with the proposed method, devoted by S_p_; trypsinization, S_t_, and the scraper method, S_s_, were lysed in SDS-PAGE sample buffer, and protein samples were extracted and analyzed by western blot (**a**). Relative protein quantities of (**b**) anti-leukemia inhibitory factor receptor (LIFR) and (**c**) integrin α5 were measured and protein quantities were normalized to β-actin. **d** Number of AP sites was measured by AP site counting kit for evaluation of DNA damage. As a positive control, some of the cells was irradiated with UV for 30 min in the extracted DNA by using a DNA extraction kit. Data are expressed as mean ± SD (*n* = 4, *p < 0.05, **p < 0.01, all *p*_SW_ > 0.05)

In conventional trypsinization, the intracellular proteins are preserved, but cell surface proteins can be disrupted^24^. We speculated that our proposed method may preserve both the surface proteins and intracellular proteins. To estimate the protein damage, we quantified β-actin, leukemia inhibitory factor receptor (LIFR) and integrin α5, extracted from cells after detachment by each method using western blotting (Fig. 3a). We selected β-actin as a stable intracellular protein for our control, and LIFR and integrin α5 to represent the surface proteins. LIFR is a cell surface receptor related to differentiation^25^. Integrin α5 is a type of integrin-transmembrane receptor that facilitates cell-extracellular matrix adhesion^26^. We compared the protein levels in cells detached by the proposed method against trypsinization and cell scraping. The control protein, β-actin, was statistically indistinguishable between the three techniques, as expected. However, both surface protein representatives, LIFR and integrin α5, were significantly reduced by trypsinization compared with the proposed method (*p* = 0.002 and *p* = 0.002, Ryan’s method). In the case of integrin α5, the reduction was likewise significant compared with the scraping method (*p* = 0.002, Ryan’s method), and lower for LIFR (Fig. 3b, c). The results suggest the digestion activity by trypsinization fail to preserve these surface proteins, while the proposed method and cell scraping manage to do so to a similar degree. In addition to the integrin α5 remaining, the initial adhesion is also improved with the proposed method. Furthermore, the focal adhesions between integrin α5 and the ECM is stronger than the adhesion between the ECM and the cell culture substrate^27^. These results indicated that the cells having ECM were detached by using the proposed method.

### DNA damage of detached cells

The number of AP (apurinic/apyrimidinic) sites of cells^28^ detached by the proposed method was equivalent to that of cells detached by trypsinization, and that of cells detached by the proposed method was smaller than that of cells irradiated with UV prepared as a positive control as shown in Fig. 3d. Hence, the cell nucleus detached by the proposed method was not damaged, similar to the observations of cells detached by trypsinization.

### Identification of detachment factors

**Fig. 4:**
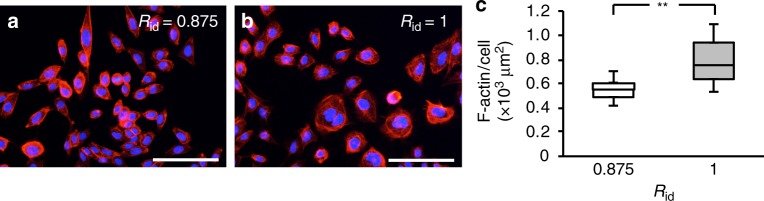
Evaluation of SFM as a potential detachment trigger in the proposed method. Cell adhesion activity was examined in cells cultured with or without SFM. **a**, **b** Cells were cultured in SSM/SFM for (**a**) immersion duration ratios *R*_id_ = 0.875 or (**b**) *R*_id_ = 1. Immunofluorescence of F-actin stained by rhodamine phalloidin (red color) and cell nucleus stained by Hoechst 33342 (blue color). **c** Comparison of the actin area in single cells cultured as indicated (*n* = 8, **p < 0.01, all *p*_SW_ > 0.05). All scale bars represent 200 µm. Boxes have the meaning of 25% and 75% quartile around the population mean value (middle line = median) and error bars indicate maximum and minimum

Because the cell detachment mechanism is key to the proposed method, we examined the factors responsible for the detachment. Detaching cells requires an initial trigger together with some form of assistance^24^, aspects we next seek to identify in the proposed method. We first confirmed the SFM’s role as the detachment trigger by determining the presence of pseudopodia via fluorescence microscopy (Fig. 4a, b) while comparing *R*_id_ = 0.875 with *R*_id_ = 1, in other words with and without SFM, respectively. The immunofluorescence data showed that F-actin of many cells in the SFM had decreased compared with cells in the SSM. We quantified the adhesion by measuring the size of the F-actin stained areas, and found that it was 29.8% greater when using the SSM alone (*R*_id_ = 1) than with the SSM/(SFM + SSM) combination (*R*_id_ = 1) (*p* = 0.009, Ryan’s method, Fig. 4c). This indicates that immersing cells in an SFM reduces the pseudopodia extension, thus encouraging cell detachment.

**Fig. 5:**
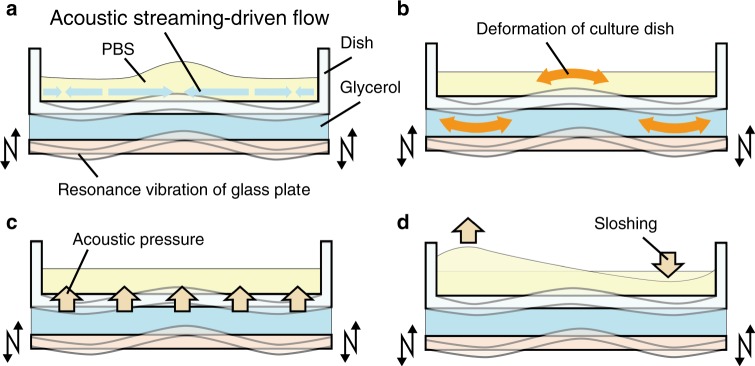
Four possible factors driving assisting cell detachment in the proposed method. **a** Shear stress from acoustic streaming-driven flow, **b** deformation of the culture dish’s base, **c** acoustic pressure, and **d** sloshing of liquid in the cell culture dish

Turning now to identify what is responsible for aiding cell detachment in our proposed method, we consider four possible mechanisms. One is the shear stress generated by acoustic streaming-driven flow^29,30^ of the PBS in the culture dish (Fig. 5a). The culture dish is caused to vibrate as well, and because the fluid volume is small, the dish’s vibration may directly be responsible for the detachment from motion at the cell–dish interface (Fig. 5b). The culture dish may also be indirectly responsible for the cell detachment, but only through the other three possible mechanisms we consider here. The vibration from the ultrasonic transducer is responsible for acoustic pressure (Fig. 5c) upon the interfaces in the system that have changes in acoustic impedance across them, and this may be a factor as the acoustic waves propagate from the dish into the adherent cells. Finally, the fluid itself may be driven into motion—sloshing—from induced vibration in the fluid volume as an acoustic cavity (Fig. 5d).

**Fig. 6:**
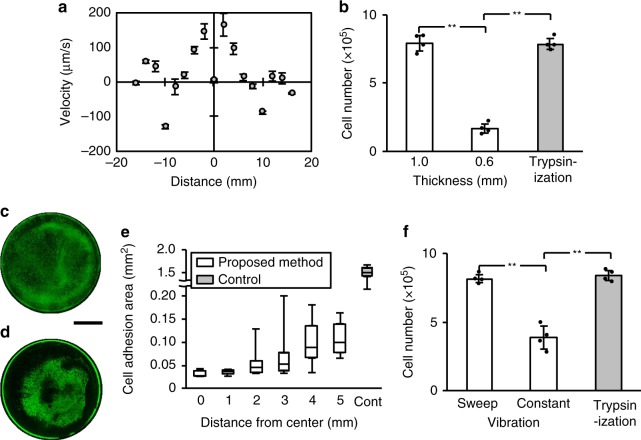
Evaluation of detachment assistance by the cell detachment system. **a** Flow velocity on the cell culture dish surface measured with 10 µm polystyrene particles. The flow velocity is along the surface with a driving frequency of 29–31 kHz and a driving voltage of 2*E*_A_; the zero position represents the center of the dish; a positive velocity here implies flow inward towards the center of the dish (mean ± SD, *n* = 50). **b** Cells were detached by either ultrasonic vibration or trypsinization. Cells were cultured in dishes with thickness of 1.0 or 0.6 mm, and the maximum vibration amplitudes of these dishes were adjusted to 0.15 µm input voltage: 2*E*_A_(mean ± SD, *n* = 4, **p < 0.01, all *p*_SW_ > 0.05). Large images of cells stained by calcein (**c**) before and (**d**) after detachment using ultrasonic vibration (input voltage: *E*_A_). A scale bar represents 10 mm. **e** Cell distribution after detachment using proposed method (*n* = 8). Boxes have the meaning of 25% and 75% quartile around the population mean value (middle line = median) and error bars indicate maximum and minimum. **f** Cells were detached by sweep or constant frequency vibration, in comparison to trypsinization. The frequency range swept by the input signal was 29–31 kHz, upward at a speed of 50 Hz from 29 to 31 kHz, then starting over at the 29 kHz value. The constant frequency signal was at 30.4 kHz, determined as the resonant frequency of the complete system at the time. The input voltage for both cases were identical at 2*E*_A_ (mean ± SD, *n* = 4, **p < 0.01, all *p*_SW_ > 0.05)

For identification of the mechanism(s) most responsible for the cell detachment, we compared the results of experiments on cell detachment caused by ultrasonic vibration (Fig. 6 and Supplementary Fig. 2), supported by numerical calculations (Supplementary Fig. 3). Figure 6a shows the cell media’s flow velocity due to acoustic streaming with respect to the distance from the dish’s center to its edge, altogether 16 mm. The effects from vibration of the cell culture dish on cell detachment was evaluated using dishes with different thickness (1.0 and 0.6 mm). Figure 6b indicates the number of cells detached by the proposed method versus trypsinization. For easier evaluation of the cell detachment area, large images of calcein-stained cells across the entire cell culture dish were observed by fluorescence microscope, as shown in Fig. 6c, d and quantified using ImageJ. Figure 6e indicates the cell distribution after ultrasonic vibration-driven detachment. In order to test the effects of sloshing of the cell media in the dish on cell detachment, the input signal to the ultrasonic transducer was either held constant at the resonance frequency—which did not cause sloshing of the cell media—or was swept over a narrow frequency range encompassing this resonance frequency—which did cause cell media sloshing. These effects are plotted in comparison to simple trypsinization as a control in Fig. 6f.

### Long-term culture and reliability of the detachment method

**Fig. 7:**
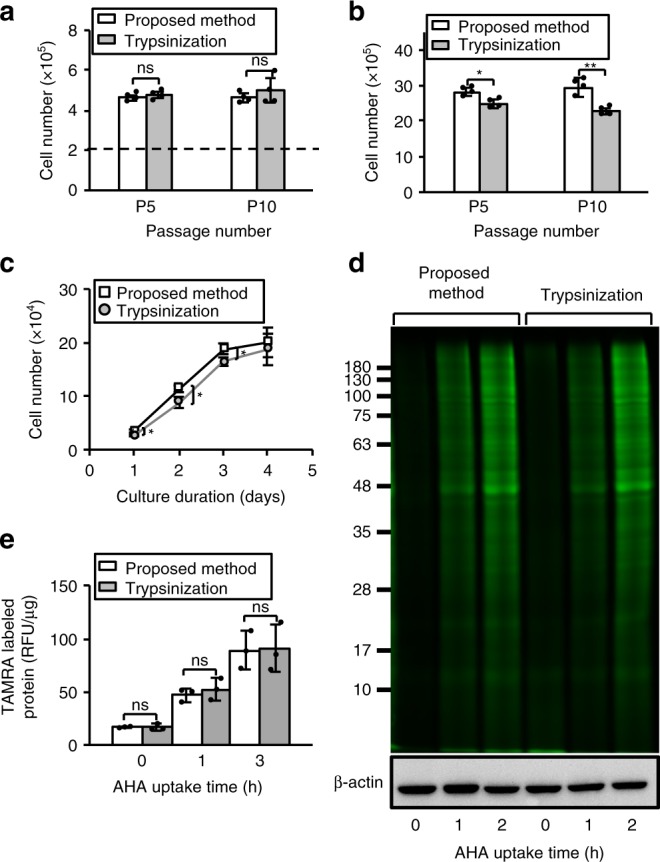
Long-term culture of CHO. **a** The number of detached cells was evaluated after 5 and 10 passages. The cells (2 × 10^5^) were seeded for culture onto a dish with *R*_id_ = 0.875, then detached either with our method or trypsinization. **b** Comparison of the cell proliferation detached with the proposed method versus trypsinization after 5 and 10 passages. **c** The growth curve of cells (1 × 10^4^) after 10 passages was measured by Cell Counting kit-8 in 96-well plate. **e**, **d** Protein productivity by the pulse chase method without radioisotope (RI). The fluorescent labeled proteins detected by tetramethylrhodamine (TAMRA)-alkyne reacted with AHA-labeled proteins were measured by (**e**) electrophoresis and (**d**) a fluorescence absorptiometer. β-Actin was observed by western blotting as a representative protein for the comparison of the amount of protein of each sample. Data are shown as mean ± SD; *n* = 4, *p < 0.05, **p < 0.01, all *p*_SW_ > 0.05

In order to confirm the reliability of the detachment method using acoustic pressure and sloshing for long-term culture of CHO cells, the number of detached cells (Fig. 7a) and the number of cells cultured for 48 h (Fig. 7b) after 5 and 10 passages, the growth curve of cells after 10 passages (Fig. 7c), and protein productivity (Fig. 7d, e) after 10 passages are evaluated. From these results, the proposed method was able to detach a similar number of cells as the method using trypsinization even after long-term culture. By approaching confluence in the 96-well plate, the number of cells detached by trypsinization in the growth curve caught up to the proposed method, however, the proliferation of cells after detachment was still improved with the proposed method. In addition, protein productivity was measured by Click-iT^®^ L-azidohomoalanine (AHA) labeling^31^. The protein productivities of cells detached by the proposed method and trypsinization had statistically indistinguishable. These results indicate that the proposed method can be used safely even for long-term culture.

## Discussion

The proposed method was able to detach 96.2% as many cells as trypsinization. In addition, the cells used in this study were able to proliferate with only the adhesive proteins they already possess: supplementation by serum was unnecessary and the proliferation was similar whether *R*_id_ = 0.875 or 1. Cells detached by the proposed method showed improved proliferative and initial adhesive properties compared with cells detached by trypsinization. These results indicate that the ultrasonic vibration did not damage the cells, and consequently cavitation is unlikely to be present. In addition, the mechanical index (MI) calculated from the pressure in PBS surrounding cells (*P*_PB_ = 3.2 × 10^5^ N/m^2^), and the average frequency (30 kHz), is 1.83. This is lower than the allowed mechanical index of 1.9 for ultrasound devices in medical applications^32^, although the details of cavitation phenomena and the threshold of its onset intimately depend upon the composition of the fluid, pre-existing cavitation nuclei, and many other parameters that the reader must keep in mind in applying this or other ultrasound-based methods. Furthermore, the proposed method also appeared to preserve cell surface receptor proteins, indicating these cells retain the ability to respond to outer signaling molecules and absorb nutrients. Since cells detached by the proposed method retain their surface proteins, the proposed method should facilitate cell adhesion in a manner superior to trypsinization. Together these findings indicate that the cells detached by the proposed method are more biologically active than cells detached by trypsinization, and suggest the proposed method is a useful improvement upon current techniques for culturing cells.

In trypsinization, trypsin functions as the detachment trigger by degrading the adhesive proteins, while pipetting and shaking serves as detachment assistance through mechanical stimulus. Cells cultured in an SFM with a low cell adhesion factor-laden extracellular matrix such as collagen and laminin show reduced cell-fibronectin and cell-cell binding ability, respectively, indicated as a shrinking of the pseudopodia as explained earlier^33^, and as determined from immunofluorescence results that showed that the area of F-actin for cells of *R*_id_ = 0.875 was smaller than for cells with *R*_id_ = 1. Together, these results indicate that the SFM is the detachment trigger.

Of the four possible mechanisms responsible for cell detachment in our system, the acoustic streaming-driven shear flow, the deformation of the culture dish itself, the acoustic pressure, and the sloshing of the liquid which drives shear flow (Fig. 5a–d), the last mechanism was determined to be the one most responsible for cell detachment.

There is an obvious steady and vertical fluid motion in the culture dish (Fig. 6a) that is characteristic of acoustic streaming (Fig. 5a)^34^. The Ekman-layer flow has a maximum shear at the center and edges of the dish. In past work, this has been shown to be responsible for agglomeration of suspended particles^35^. However, ultimately it was found that the maximum acoustic streaming-driven flow velocity in the system was 150 µm s^−1^, about three orders of magnitude less than what is required to detach the cells, about 89–400 mm s^−1^ (see Eqs. [1–3] in the Supplementary Information). Hence, acoustic streaming is present but not responsible for the observed cell detachment.

The deformation of the culture dish’s base (Fig. 5b) is also potentially a reason for detachment of the cells through the acceleration of the substrate they are adherent upon. In an attempt to separate the effects, two different culture dishes were used, one with a dish thickness of 1 mm, the other 0.6 mm. The thinner dish is far easier to vibrate, and we set out to adjust the amplitude of the input signal to the piezoelectric element so that the amplitudes of vibration of the two dishes were equal at 0.15 µm at 30.4 kHz (Supplementary Fig. 2c, d). In doing so, the number of cells released by the two plate choices were significantly different: the thicker 1 mm dish was still effective in releasing cells, but the 0.6 mm dish was not, only releasing 17.6% as many cells as shown in Fig. 6b (*p* = 0.00001, Ryan’s method). The fact the detachment results are different between the two dish thicknesses indicate the deformation of the culture dish during vibration is not directly responsible for the observed cell detachment: identical dish vibration produces different detachment results. The indirect effect of this change in causing different acoustic streaming, acoustic pressure, or other effects may be important, but the point here is that the dish’s vibration itself is not. As mentioned before, the indirect effects are already taken into consideration via the other three possibilities.

The acoustic radiation force is a typical consequence of the acoustic pressure delivered across an interface (Fig. 5c). The maximum acoustic radiation pressure upon the cells from the dish was calculated (see Eqs. [4, 5] in the Supplementary Information) for both traveling and standing acoustic waves to produce an upper bound for these quantities. The maximum acoustic radiation pressure for traveling wave (6.74 × 10^−27^ N m^−2^) and standing waves (4.93 × 10^−18^ N m^−2^) were extremely small and Christakou et al. reported that the acoustic radiation force was insufficient for cell detachment with a microarray device with ultrasonic irradiation^36^, suggesting the acoustic radiation pressure is not a factor in cell detachment. However, because *w*, suggesting the acoustic radiation pressure is not a factor in cell detachment. However, because we used a frequency-swept input in our experiments, the generated acoustic field is not constant (Supplementary Movie 1), and this transient phenomenon may cause a greater acoustic pressure upon the cells. To determine whether this is the case, the difference in acoustic pressure between the upper and lower faces of the attached cells was computed (see Eqs. [6–8] in the Supplementary Information), assuming an intermittent traveling wave with attenuation was present that would produce the greatest possible acoustic radiation pressure in our system. The maximum acoustic pressure difference in the media from beneath to above a given cell was found to be ~38.8 N/m^2^, showing a far greater acoustic pressure and indicating the importance of taking into account the transient nature of the excitation in our system. The pressure required to detach cells in past research is stated to be about 10–100 N m^−2^ ^37^, though in the presence of SFM the adhesion of the cells is expected to be reduced from these values. Further, the acoustic impedance of the culture dish (=2.52 × 10^6^ kg m^−2^ s^−1^ ^38^) is higher than the PBS media (=1.565 × 10^6^ kg m^−2^ s^−1^ ^39^) and the acoustic pressure caused by the intermittent traveling wave is generated in the system from vibration of the culture dish. This means the edges of the filopodia of the cells adhering to the culture dish receive a greater acoustic pressure than our simple PBS-surrounded cell calculations would suggest, and so the actual acoustic pressure experienced by the cells during the intermittent, traveling-wave ultrasound could easily be >38.8 N m^−2^. Notably, cells remain attached near vibration nodes of the dish, detaching in antinodal locations (Supplementary Fig. 2c) at the dish’s periphery and center (Fig. 6c–e). Consequently, the acoustic pressure caused by the intermittent traveling wave is important to the observed cell detachment. However, the number of cells detached from the culture dish when increasing its thickness from 0.6 to 1.0 mm was increased as seen in Fig. 6b even though the vibration amplitude of the culture dishes were nearly identical (see Supplementary Fig. 2c, d). This indicates that the acoustic pressure caused by the intermittent traveling wave contributes to the detachment but is not the sole contributor.

Another potential reason for the observations could be fluid sloshing^40^ (Fig. 5d), where a resonant mode is driven in the fluid cavity, causing either radial, axisymmetric fluid motion or antisymmetric fluid motion with sloshing back and forth across the dish. The horizontal fluid motion responsible for the shear in this arrangement becomes small near the periphery of the disk, regardless of whether the motion is axisymmetric or not, and so the cells would detach from this location relatively poorly. Also, the vibration should likewise give rise to a measurable vertical oscillation of the fluid-air interface as a linear response to the excitation sweep frequency of 29–31 kHz: at a given location upon the dish, the fluid depth would wax and wane with fluid flows across the dish. The measured horizontal fluid motion (Fig. 6a) does have a distribution matching what one would expect with sloshing, although the fluid velocity at the center is zero, indicating that if there is sloshing, it exhibits a radial symmetry. Observation of the fluid free surface to a thousand frames per second indicated evidence of sloshing (Supplementary Movie 1). From the vertical motion of the fluid surface, an estimate of the in-plane sloshing flows was determined, from which the shear upon the adherent cells was calculated (see Eqs. [9–11] in the Supplementary Information). The maximum velocity of the shear flow was found to be ~14.9 mm s^−1^. Although this value is smaller than the value believed to be necessary for cell detachment through shear flow (89–400 mm s^−1^), it is sufficient in consideration of the use of the SFM and the influence of the acoustic pressure to be a factor in the detachment process. Most importantly, far more cells were detached by using sweep vibration—with sloshing as a consequence—than sloshing-free constant vibration, as shown in Fig. 6f. From this evidence, sloshing is an important factor in cell detachment, and together with the transient effects of the acoustic radiation pressure, it contributes to the favorable cell detachment results seen in this study.

Therefore, the mechanism responsible for aiding cell detachment is the acoustic pressure and the sloshing caused by the intermittent traveling wave. It gives rise to sufficient pressure and shear flow to detach the cells from the substrate if the SFM is present as the detachment trigger. It is noteworthy that the maximum pressure difference between PBS above and below the cell is close to the pressure necessary to detach cells, while the sloshing shear flow from sweep vibration likewise is sufficient to support cell detachment. Without the SFM, only 69.5% of cells were detached, substantially inferior to the combination of the SFM and the intermittent traveling-wave device which detached 96.2% as many cells as trypsinization. In addition, cells detached by the proposed method had greater proliferative activity than the cells detached by conventional trypsinization, and the cell surface proteins exhibited less damage with the proposed method than with trypsinization. From the number of AP sites of cells, cell culture with the proposed method has no damage to cellular DNA. Even in long-term culture, the proposed method demonstrates a good detachment ratio and proliferation comparable to single detachment. The results from long-term culture show no damage to protein productivity after 10 passages. Supplementary Fig. 5 also indicates that not only CHO cells but also several cell types including human/non-human and cancer/non-cancer cell lines were able to be detached efficiently with the proposed method. Thus, we conclude that a remarkably effective enzyme-free cell detachment method that produces results superior to conventional trypsinization is made possible through a combination of the three effects together: acoustic pressure of the intermittent traveling wave, sweep signal-driven sloshing, and the use of an SFM.

Our previous cell detachment methods using resonance vibration of the culture base with enzyme^41,42^ resulted in less damage to the cells than trypsinization, but an enzyme such as trypsin was always used for the detachment trigger, leaving the problem of cellular damage unsolved. Likewise, temperature modulation produces less damage^24^, but the cell proliferation was significantly decreased from the cooling required in this method. Other groups have found that the use of SAW devices can generate sufficient acoustic flow to detach cells, and cell detachment methods with SAW devices have been reported^43,44^. However, many SAW devices were required to print the IDT fingers directly onto the cell culture substrate, due to strong attenuation of the high-frequency MHz ultrasound used in these devices. There is another study using the indirect irradiation of ultrasound from SAW device via PDMA^45^, but the cell detachment area was sparse and narrow. Hence, these SAW device methods are unsuitable for cell detachment with the standard culture dish typical in current clinical and laboratory practices.

By contrast, the proposed method eliminates the detachment enzymes and concomitant cellular damage, does so in a way compatible with current clinical and laboratory practices, even when scaled to industrial high-throughput systems, and occurs via a well-characterized combination of the acoustic pressure and the sloshing caused by the intermittent traveling wave and the use of SFM. The proposed method also can detach cells using standard dishware and the cell detachment was reliable even for cells cultured over long terms, so the method is appropriate in medical applications. We anticipate that by adopting this method, cell culture operations can proceed with greater throughput, efficiency, and cell and cell product quality.

## Methods

### Experimental setup

The cross-section image of the cell detachment system is shown in Supplementary Fig. 1. The system is mainly composed of an ultrasonic transducer that consists of a glass plate made of Soda-lime glass and a piezoelectric ceramic ring (HC-52R23, Honda Electronics Co., Ltd., Tokyo, Japan), a felt (GY00176, YoSoo, Hong Kong), a silicone rubber (KE-1316, Shin-Etsu Chemical Co. Ltd., Tokyo, Japan), two polycarbonate bases (Acrylic shop Hazaiya, Tokyo, Japan), glycerol (17029-00, Kanto Chemical Co., Tokyo, Japan), a ø35 cell culture dish (thickness: 1.0 mm, 3000-035, Tissue culture dishes, AGC Techno Glass, Shizuoka, Japan, and thickness: 0.6 mm, 430165, Cell culture dish, Corning Inc, NY, USA), and a weight made of stainless steel (100 g). Note that the glass plate and the piezoelectric ceramic disk are bonded by epoxy adhesive (ARALDITE RT30, Araldite, Huntsman Corporation, TX, USA). In addition, the ultrasonic transducer, the felt, the silicone rubber and two polycarbonate bases are clumped by M3 bolts and nuts hand-tight. The dimensions of the glass plate, the piezoelectric ceramic ring and the cell culture dish are shown in Supplementary Table 1. Note that, the height of the gap filled with glycerol between the glass plate and the cell culture dish is 0.5 mm, and the depth of PBS is ~2 mm when 2 mL PBS is introduced. The vibration on the glass plate is excited by applying sweep AC input that is generated by a function generator (WF1974, NF Corporation, Kanagawa, Japan) and an amplifier (HSA 4051, NF Corporation) to the piezoelectric ceramic ring. The diameter and the depth of the cell culture dish are 35 and 11 mm, respectively.

### Measurement of vibration characteristics

Supplementary Fig. 2a shows the resonance vibration mode of the glass plate, which has two nodal circles. From the measurement results of the vibration amplitude distribution of the resonance vibration, which is excited on the device with a resonance frequency of 30.4 kHz and an input voltage of 100 V, the excited vibration mode clearly corresponds to the out-of-plane vibration mode with two nodal circles whose radius are around 5 and 13 mm. Note that, we measured vibration amplitude distribution from the center of the cell culture dish to ±16 mm. We obtained vibration amplitude distribution using a laser Doppler vibrometer (LV1800, Ono Sokki Co. Ltd., Kanagawa, Japan). Note that the vibration amplitude of the glass plate was measured while the cell culture dish (thickness: 1.0 mm) was mounted on the glass plate. Glycerol was placed to fill the gap between the glass plate and the cell culture dish. Supplementary Fig. 2b shows the relationship between input voltage and maximum amplitude at the center of the glass plate at a resonance frequency—30.4 kHz. We furthermore measured the vibration amplitude distribution of the cell culture dishes at that time, finding, as shown in Supplementary Fig. 2c, d, that the vibration distribution of the cell culture dishes were essentially identical to the vibration distribution of the glass plate. This indicates that while the dishes themselves may have different vibration modes and behaviors due to differences in their thickness and other factors, they respond in a manner matching the driven shape of the glass plate. The vibration amplitude distributions of the cell culture dishes with the thickness of 1.0 and 0.6 mm are shown in Supplementary Fig. 2c, d, respectively. The maximum vibration amplitude of the culture dish with a thickness of 0.6 µm (input voltage: 36 V) was adjusted to match the amplitude produced by the 1.0-µm thick dish (input voltage: 200 V). Because of how they both respond to the excitation via the vibration of the glass plate, note that the mode shapes of the two dishes are nearly identical; the implication is that the dish is responding passively to the driven vibration energy provided via the coupling fluid from the transducer.

### Cell culture

The Chinese hamster ovary CHO cell line (CHO-K1, RCB0403, Riken Bio Resource Center, Ibaraki, Japan) was used as a representative adherent cell line, as CHO cells are commonly used for protein production^46^. The CHO cells were cultured in serum-supplemented Ham’s F-12 medium (α-MEM Ham’s F-12, Wako, Tokyo, Japan) supplied with 10% fetal bovine serum (FBS, S1820, Biowest SAS, Nuaillé, France) in a 5% CO_2_ humidified atmosphere incubator at 37 °C. Cell passage was performed by trypsinization with 0.050% trypsin-EDTA (25300, Life Technologies, CA, USA) by pipetting.

The CHO cells were passaged the minimal number of times needed for experiments and were seeded in cell culture dishes. Cells seeded in the 2 mL SSM were incubated for 24–48 h in a 5% CO_2_ humidified atmosphere incubator at 37 °C and then used for cell detachment experiments.

#### Proposed method and trypsinization

We detached cells with the proposed method using ultrasonic vibration after treatment of SFM. CHO cells were first seeded on the cell culture base and cultured in SSM for a specific incubation time. The medium was removed and then cells were cultured with the SFM with Insulin-Transferrin-Selenium Liquid Media Supplement (100×) (Sigma-Aldrich, MO, USA) for a specific incubation time. Cells were washed three times with PBS dissolved in DI water, and then PBS (2 mL) was spread around the chamber. The CHO cells were then exposed to ultrasonic vibration for detachment. Note that the sinusoidal signal input to the ultrasonic transducer was swept from 29 to 31 kHz, bracketing the resonance frequency of the dish (Supplementary Fig. 2). The cells were rinsed and aspirated from the culture chamber by a micropipette for collection.

In conventional trypsinization, CHO cells were detached by exposure to 0.050% trypsin-EDTA for 5 min at 37 °C and then collected by pipetting. In the first set of experiments to determine vibration conditions (Fig. 2a), cells were immersed in the SFM for 24 h before trypsinization (the same duration as the proposed method) for matching the number of adhered cells to the same before detachment. In the other experiments (Figs. 2b–6), cells were only cultured in the SSM before trypsinization as a control.

### Cell detachment assay

The cell detachment methods (proposed method and trypsinization) are briefly outlined in the Methods section. The number of seeded cells were 4.0 × 10^5^ and 2.0 × 10^5^ in Fig. 2a, b, respectively. The numbers of cells detached by the proposed method and trypsinization were counted by a hemocytometer (A116, Asone, Osaka, Japan) under phase contrast microscopy (ECLIPSE Ti, Nikon Corporation, Tokyo, Japan), and this process was repeated four times. The number of live cells was measured with Trypan Blue Solution (0.4%) (Thermo Fisher Scientific, MA, USA) on a hemocytometer. Note that the driving voltage was defined as *V*_A_ = 100 V, and the immersion duration ratios, *R*_id_ = (time while exposed to SSM)/(time while exposed to SSM and SFM). The total cell culture duration was always fixed at 48 h: in other words, SSM h + SFM h = 48 h always.

### Cell proliferation assay

The cells were detached by the proposed method or by trypsinization, and then cells (8.0 × 10^5^) were reseeded in 60 mm dishes (AGC Techno Glass Co. Ltd.) and incubated for 24 or 48 h. Cells were treated with trypsin for 10 min and then counted with a hemocytometer. Cell counting was repeated four times.

### Initial adhesion assay

The cells were detached by the proposed method or trypsinization, and then cells (2.0 × 10^5^) were reseeded in 35 mm dishes (AGC Techno Glass Co. Ltd.) and incubated for 5 min. To evaluate the number of initially adhered cells, the medium was collected and the remaining adherent cells were trypsinized for 10 min and counted by a hemocytometer. Cell counting was repeated four times. We further observed the initially adhered cells using a scanning electron microscopy (SEM, S-4700, Hitachi High-Technologies Corporation, Tokyo, Japan). To evaluate cells in detail, the cells were freeze-dried with *t*-butyl alcohol and observed.

### Protein quality assay

CHO cells were lysed in SDS-PAGE sample buffer (7.0 × 10^5^ cells/500 µL) and then heated at 70 °C for 5 min. Protein samples (5 µL for integrin α5 analysis or 10 µL for leukemia inhibitory factor receptor (LIFR) analysis) were loaded in each lane and resolved on SDS-PAGE (7.5% Tris-HCl gels), followed by transfer onto polyvinylidene fluoride membranes. The membranes were gently incubated in a blocking solution, Blocking One (Nacalai Tesque, Kyoto, Japan), for 1 h. The membranes were incubated with the following primary antibodies at 4 °C for 16 h: 0.4 µg/mL rabbit anti-human LIFR polyclonal antibody (22779-1-AP; Proteintech, Rosemont, IL, USA), a 1000-fold dilution of rabbit anti-human Integrin α5 monoclonal antibody (#98204; Cell Signaling Technology, Boston, MA, USA), and a 1000-fold dilution of rabbit anti-mouse β-actin polyclonal antibody (#4967; Cell Signaling Technology). The membranes were washed in TBS containing 0.05% Tween 20 (TBS-T) for 10 min for three washes. The membranes were incubated with a horseradish peroxidase (HRP)-labeled secondary antibody (1:10,000 dilution, HRP-conjugated anti-rabbit IgG; Perkin-Elmer, Waltham, MA, USA) for 30 min. After three washes of membranes by TBS-T for 10 min each, the membranes were treated with chemiluminescent reagent (Immobilon Western, Millipore). Finally, the membranes were analyzed by an image analyzer (Fluoro Chem Q, Protein Simple). The band density was normalized with the β-actin band and was expressed as the quantity relative to trypsinization. Note that the scraping method (S_s_) described in Fig. 3 is used as a control in quantifying the surface protein present upon the cells, and was conducted by scraping the entire surface of the culture dishes with a cell scraper (TPP cell spatula 99010, Techno Plastic Products AG, Trasadingen, Switzerland). Nate that, full gel images were shown in Supplementary Fig. 4.

### DNA damage assay

DNA was extracted from the cells detached by the proposed method or trypsinization after 10 passages. A DNA extraction kit (Get *pure*DNA Kit-Cell, Tissue, Dojindo, Tokyo, Japan) was used to extract the DNA. In the extracted DNA, some of the cells detached by trypsinization was irradiated with UV (Mineralight XX-15S, Analytic Jena, Jena, Germany) for 30 min as a positive control. In order to evaluate the DNA damage, the number of apurinic/apyrimidinic (AP) sites in the DNA in the cells detached by either the proposed method, trypsinization, or UV irradiation was measured by using an AP site counting kit (-*Nucleostain*- DNA Damage Quantification Kit, Dojindo, Tokyo, Japan).

### Adhesion area assay

Cells were immersed in the SSM/SFM for *R*_id_ = 0.875 or 1. Cells were fixed with 2.5% glutaraldehyde, and F-actin and cell nuclei were stained by Rhodamine Phalloidin (R415, Thermo Fisher Scientific) and Hoechst 33342 (Thermo Fisher Scientific), respectively. Cells were observed by a fluorescence microscope (ECLIPSE Ti-S, Nikon, Tokyo, Japan). The area of F-actin in each cell was measured by ImageJ (National Institutes of Health, Bethesda, MD).

### Detachment capacity on dishes with different thickness

The influence of the vibration of the cell culture dish was evaluated using dishes with different thickness. We examined dishes with 1.0 and 0.6 mm in thickness. Cells were detached by the proposed method or trypsinization. The conditions of vibration characteristics (frequency = 29–31 kHz, input voltage = 2*E*_A_ (*E*_A_ = 100 V), sweep cycle = 10 ms, and exposure duration = 5 min) and cell experiment procedures (*R*_id_ = 0.875) were similar to the cell detachment assay. Note that the maximum amplitudes of dishes of 0.6 mm thickness (Supplementary Fig. 2d) were adjusted to be equal to the maximum amplitude of dishes of 1.0 mm thickness (vibration amplitude of the center of dish = 0.15 µm). The number of live cells was measured with Trypan blue using a hemocytometer.

### Detachment capacities of sweep and continue vibration

The influence of the sweep vibration of the cell culture dish was evaluated using dishes with different thickness. We examined the sweep vibration and the continue vibration. Cells were detached by the proposed method or trypsinization. The conditions of the sweep vibration were to use a frequency range of 29–31 kHz, an input voltage of 2*E*_A_, an upward and a downward speed of 50 Hz, and an exposure duration of 5 min. For the continuous vibration experiment, the resonance frequency was identified during the experiment, and a frequency of 30.4 kHz was used with an input voltage of 2*E*_A_ and exposure duration of 5 min. Note that cell experiment procedures (*R*_id_ = 0.875) were similar to the cell detachment assay. The number of live cells was measured with Trypan blue using a hemocytometer.

### Detachment capacity of four factors

In the relationship between the distance from the center to the edge of dish (16 mm) and flow velocity, the flow velocity was measured by taking a movie of polystyrene particles (diameter: 10 µm; Micromer, Micromod Partikeltechnologie GmbH, Rostock, Germany) and by analysis using ImageJ. The conditions of vibration characteristics were adjusted: frequency: 29–31 kHz, input voltage: 2*E*_A_, upward and downward speed: 50 Hz, and exposure duration: 5 min. Cells were immersed in the SSM/SFM for *R*_id_ = 0.875, stained by calcein (Calcein-AM, Thermo Fisher Scientific) for 30 min and, observed before and after the detachment. The detachment conditions of vibration and cell experiments were adjusted: frequency: 29–31 kHz, upward and downward speed: 50 Hz, exposure duration: 5 min, input voltage: *E*_A_, and *R*_id_: 0.875. Large images of cells stained by calcein on the entire cell culture dish were observed by the fluorescence microscope. In order to remain a part of cells on the culture surface after detachment, the input voltage was identical at *E*_A_, and cell distribution after the detachment using ultrasonic vibration as shown in Fig. 6e. Area of the cells stained by calcein was measured using ImageJ. In Fig. 6f, the frequency range swept by the input signal was 29–31 kHz, upward at a speed of 50 Hz from 29 to 31 kHz, then starting over at the 29 kHz value. The constant frequency signal was at 30.4 kHz, determined as the resonant frequency of the complete system at the time. The input voltage for both cases were identical at 2*E*_A_.

### Fluid surface motion recorded by high-speed camera

In order to check the effect of the ultrasonic vibration on the fluid surface, the fluid surface motion was recorded by high-speed camera (VW-6000, Keyence, Osaka, Japan). The movie was recorded from above at 30° from the vertical in order to visualize the motion via specular reflection of light from the fluid interface. The movies captured by high-speed camera was 1000 fps, and the provided movie is encoded to play at 15 fps.

### Long-term culture

The CHO cell line (CHO-K1, RCB0403, Riken Bio Resource Center, Ibaraki, Japan) was used for long-term culture. The CHO cells were cultured in serum-supplemented Ham’s F-12 medium (α-MEM Ham’s F-12, Wako, Tokyo, Japan) supplied with 10% fetal bovine serum (FBS, S1820, Biowest SAS, Nuaillé, France) and 1% antibiotics (antibiotic-antimycotic mixed stock solution (×100) Nacalai tesque, Kyoto, Japana) in a 5% CO_2_ humidified atmosphere incubator at 37 °C. A total of 10 passages with the proposed method and trypsinization were performed before reaching confluence, respectively.

CHO cells were first seeded on the cell culture base and cultured in serum-supplemented medium (SSM) for 42 h. The medium was removed and then cells were cultured with the SFM with Insulin-Transferrin-Selenium Liquid Media Supplement (100×) (Sigma-Aldrich, MO, USA) for 6 h. That is, the immersion duration ratios, *R*_id_ = 0.875. Cells were washed three times with PBS dissolved in DI water, and then PBS (2 mL) was spread around the chamber. The CHO cells were then exposed to ultrasonic vibration for detachment. Note that the sinusoidal signal input (input voltage: 2*E*_A_) to the ultrasonic transducer was swept from 29 to 31 kHz, bracketing the resonance frequency of the dish. The cells were rinsed and aspirated from the culture chamber by a micropipette for collection. In conventional trypsinization, CHO cells were detached by exposure to 0.050% trypsin-EDTA for 5 min at 37 °C and then collected by pipetting.

The CHO cells were passaged at most three times before the experiments and were seeded in cell culture dishes. Cells seeded in the 2 mL SSM were incubated for 48 h in a 5% CO_2_ humidified atmosphere incubator at 37 °C and then used for cell detachment experiments.

### Cell detachment assay

The cell detachment methods (proposed method and trypsinization) are almost the same as cell detachment assay. The number of seeded cells was 2.0 × 10^5^ in Fig. 7a. The numbers of cells detached by the proposed method and trypsinization after 5 and 10 passages were counted by a hemocytometer (A116, Asone, Osaka, Japan) under phase contrast microscopy (ECLIPSE Ti, Nikon Corporation, Tokyo, Japan), and this process was repeated four times. The number of live cells was measured with Trypan Blue Solution (0.4%) (Thermo Fisher Scientific, MA, USA) on a hemocytometer (A116, Asone, Osaka, Japan).

### Cell proliferation assay

The cells were detached by the proposed method or by trypsinization, and then cells (8.0 × 10^5^) were reseeded in 60 mm dishes (AGC Techno Glass Co. Ltd., Shizuoka, Japan) and incubated for 48 h after 5 and 10 passages. Cells were treated with trypsin for 10 min and then counted with a hemocytometer. Cell counting was repeated four times. In addition, the growth curve of CHO cells after 10 passages was measured using a cell counting kit (341-07761, Dojindo, Tokyo, Japan). The detached cells (1.0 × 10^4^) suspended with 100 mL SSM were seeded on a 96-well plate (167008, Thermo Fisher Scientific, MA, USA) and incubated for 1–4 days. In the measurement of cell number, 10 μL Cell Counting kit-8 was added and incubated for 2 h, and then the absorbance at 450 nm was measured with a spectrophotometer (Multiskan FC Basic, Thermo Fisher Scientific, MA, USA).

### Pulse chase method without radioisotope (RI)

In order to measure the proteins produced in cells, the pulse chase method without RI was performed using Click-iT^®^ AHA (L-azidohomoalanine) (C10102, Thermo Fisher). In the pulse chase method without RI, cells were cultured for 1 h in a medium without methionine, which was necessary to produce all proteins, and all proteins production were once stopped. Adding Click-iT^®^ AHA, the cells started to make the AHA containing proteins. The produced proteins were fluorescence-labeled by adding tetramethylrhodamine (TAMRA)-alkyne through click chemistry. The labeled proteins were analyzed by electrophoresis and a fluorometer.

For evaluation of the protein productivity, cells detached by either the proposed method or trypsinization were cultured to 80–90% confluence. The cells were washed by PBS and cultured in a methionine-free medium (Nutrient mixture Ham’s F-12 w/L-Glutamine w/o Methionine, Cysteine, United States Biological, MA, USA) for 1 h. Cells were treated with 50 µM AHA for 0 (immediately collected), 1 and 2 h and collected by cell scraper (Cell Scraper S, TPP Techno Plastic Products AG, Trasadingen, Switzerland). Then the collected cells were lysed in Tris-HCl (pH 8.0)/1% SDS. Fluorescence was labeled in Click-iT protein reaction buffer (C10276, Thermo Fisher) according to the manufacturer’s instruction. In brief, TAMRA-alkyne (T10183, Thermo Fisher) (40 µM) in Click-iT^®^ reaction buffer was added to AHA-labeled cell lysates. Subsequently, 40 mM CuSO_4_ was added to the mixtures to promote click reaction. Then Click-iT^®^ reaction buffer additive 1 solution was added to the mixture. After the reaction, Click-iT^®^ reaction buffer additive 2 solution was added to each mixture and then the mixtures were vortexed to complete click reaction. After that, non-labeled TAMRA was removed. To quantify TAMRA-labeled proteins, the fluorescence of TAMRA was measured by using microplate reader Flexstation3 (Ex: 545 nm Em: 580 nm). Also, the protein concentrations were measured by BCA assay kit according to instructions (Bio-Rad). Florescence intensities were normalized by the protein quantities. Further, TAMRA-labeled proteins (6.25 µg/lane) were analyzed using SDS-PAGE (10% Tris-HCl gels). TAMRA-labeled proteins were detected using FluorChemQ image analyzer. The entire proteins were stained by Coomassie brilliant blue (CBB) as shown in Supplementary Fig. 5. Furthermore, the quantities of β-actin detected by western blotting were used as the loading controls (Fig. 7d).

### Culturing of multiple cell types

In addition to CHO cells, a mouse myoblast, C2C12, a human mesenchymal stem cell (MSC), UE7T and a human cervical cancer cell, HeLa were detached by the proposed method and trypsinization. The basic experimental conditions were the same as CHO experiments, but the SSM of C2C12, UE7T and HeLa were D-MEM (Dulbecco’s modified Eagle’s medium/Nutrient Mixture F-12 Ham, Sigma-Aldrich) with 10% FBS, α-MEM (2144-05, Nacalai tesque) with 20% FBS and MEM (Minimum Essential Medium Eagle, Sigma-Aldrich) with 10% FBS, respectively. The SFM were changed from FBS of these SSM to an Insulin-Transferrin-Selenium supplement. In the cell detachment assay, 2.0 × 10^5^ seeded cells were used in Supplementary Fig. 6a. In the cell proliferation assay (Supplementary Fig. 6b), the cells were detached by the proposed method or by trypsinization, and then (8.0 × 10^5^) cells were reseeded in 60 mm dishes and incubated for 48 h. Cells were treated with trypsin for 10 min and then counted with a hemocytometer.

### Statistics and reproducibility

Samples were performed using analysis of variance (ANOVA) with Ryan’s multiple comparison test. A value of *p < 0.05 or **p < 0.01 was considered significant. Normality of each data set was assessed using the Shapiro–Wilk test *p*-value (*p*_SW_ > 0.05), and all reported data sets were found to have normal distributions based on this test.

### Reporting summary

Further information on research design is available in the Nature Research Reporting Summary linked to this article.

## Supplementary information

Supplementary Information

Description of Additional Supplementary File

Supplementary Data 1

Supplementary Movie 1

Reporting Summary

## Data Availability

All data supporting the findings of this study are available in the Supplementary Information or Supplementary Data 1 that contains the source data underlying main figures.
