# Supplementary material for: Enzyme-free release of adhered cells from standard culture dishes using intermittent ultrasonic traveling waves
Source: Commun Biol. 2019 Oct 29;2:393. doi: 10.1038/s42003-019-0638-5 (PMC6820801; doi:10.1038/s42003-019-0638-5)
Supplement: Supplementary file 1 — Supplementary Information [file 42003_2019_638_MOESM1_ESM.pdf]

Supplementary Figures

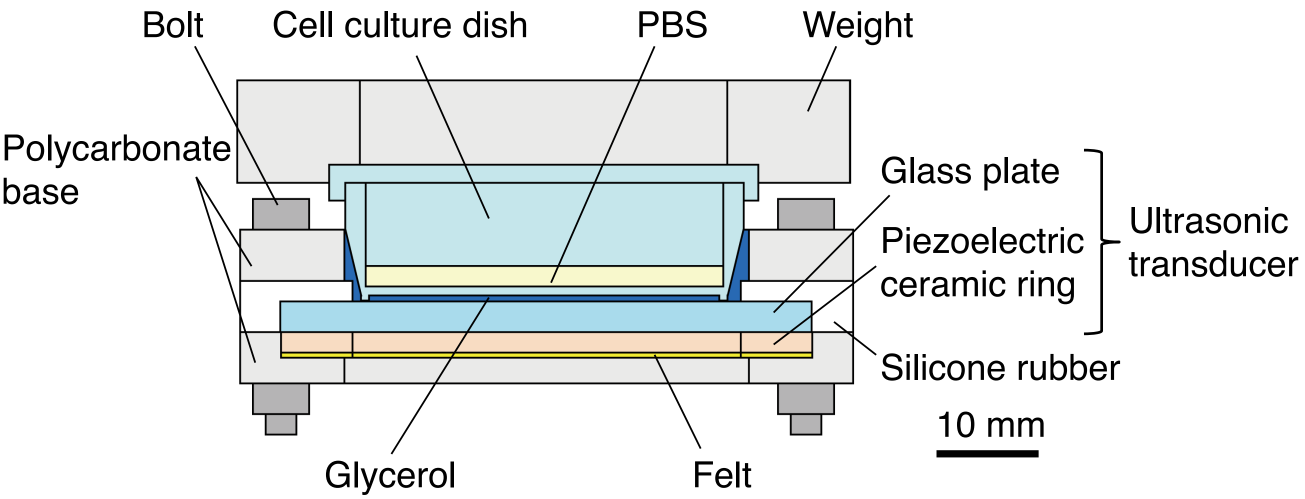

Supplementary Figure 1 | Schematic illustration of the cross-section showing the cell detachment system. The ultrasonic transducer is composed of a glass plate and a piezoelectric ceramic ring glued to the glass plate. The image is drawn to scale.

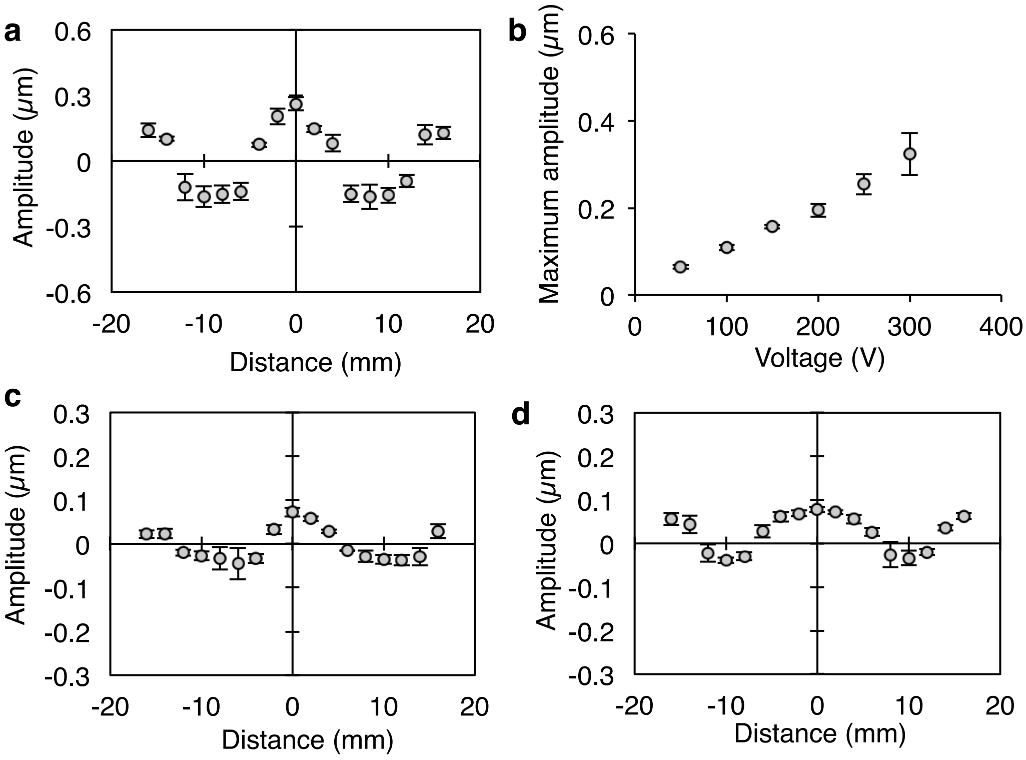

Supplementary Figure 2 | Vibration mode measured by laser Doppler vibrometer. (**a**) The vibration amplitude along the glass plate surface with a driving frequency of 30.4 kHz and a driving voltage of 100 V; the zero position represents the center of the glass plate. (**b**) The relationship between input voltage and maximum amplitude in the center of a culture dish with a driving frequency of 30.4 kHz. The vibration amplitude along a cell culture dish with (**c**) 1.0 mm thickness and (**d**) 0.6 mm thickness with a driving frequency of 30.4 kHz and a driving voltage of 200 and 36 V, respectively; the zero position represents the center of the dish (mean ± SD, with *n* = 4 trials).

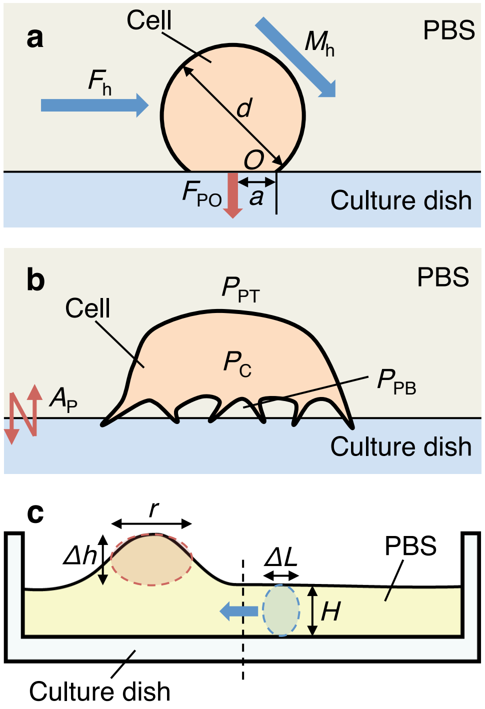

Supplementary Figure 3 | Numerical modeling of cell detachment. (**a**) The rolling detachment model caused by acoustic streaming. (**b**) The pressure detachment model caused by acoustic pressure of intermittent traveling wave. (**c**) The shear flow detachment model caused by sloshing of sweep vibration.

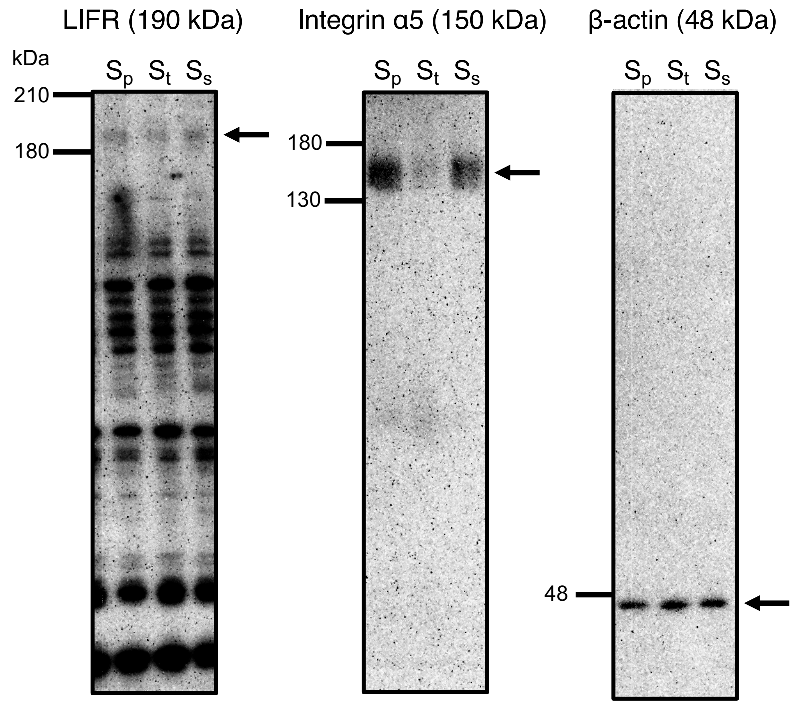

Supplementary Figure 4 | Full gel images of Fig. 3a.

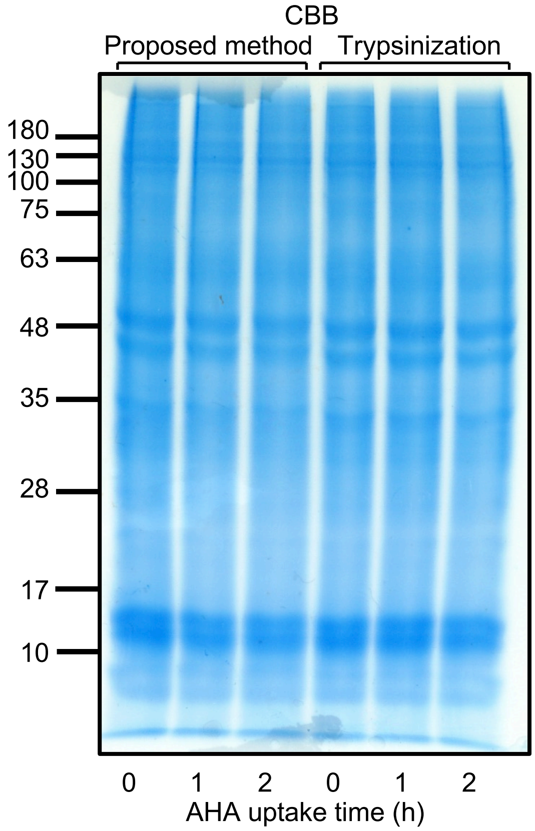

Supplementary Figure 5 | Images of electrophoresis for normalization of evaluation of protein productivity. The total amount of proteins in cells was observed by electrophoresis in Coomassie brilliant blue (CBB).

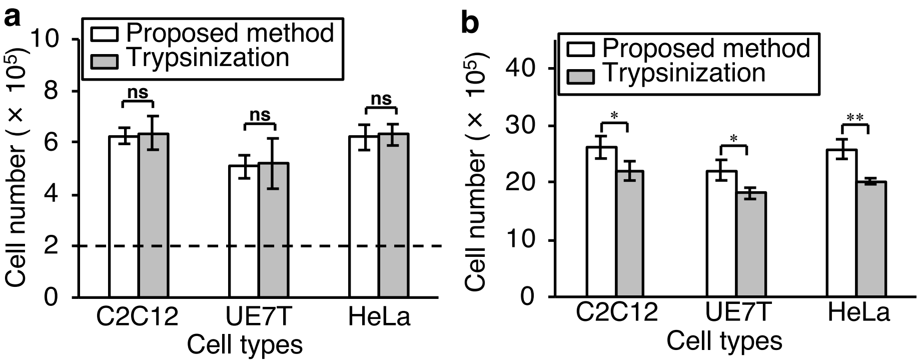

Supplementary Figure 6 | Detachment of multiple cell types. (**a**) Cell detachment ratio and (**b**) cell number 48 h after reseeding using three different types of cell lines (a mouse myoblast—C2C12, a human mesenchymal stem cell (MSC)—UE7T and a human cervical cancer cell—HeLa). Data are shown as mean ± SD; $n = 4$, *$p < 0.05$, **$p < 0.01$, all $p_{SW} > 0.05$.

Supplementary Table 1 | The dimension parameters of the cell detachment system shown in Supplementary Figure 1.

	Diameter (mm)		Thickness (mm)
	Inner	Outer	
Glass plate	52		3
Piezoelectric ceramic ring	38	52	2
Cell culture dish	35	40	1 (Bottom)

Supplementary Notes

Numerical analysis of cell detachment

In order to identify the dominant detachment factor from acoustic streaming, intermittent traveling wave, acoustic radiation pressure, and sloshing, we performed an analysis for three simple detachment models (Supplementary Fig. 3). These are the rolling detachment model caused by acoustic streaming (Supplementary Fig. 3a), the pressure detachment model caused by intermittent traveling wave (Supplementary Fig. 3b), and the shear flow model caused by sloshing from sweep vibration (Supplementary Fig. 3c).

First of all, we calculated the shear velocity caused by acoustic streaming necessary for cell detachment. In the detachment rolling model (Supplementary Fig. 3a), the relationship of the hydrodynamic torque (M_{h}), the hydrodynamic drag force (F_{h}) and the pull-out force to detach (F_{po})[1] is

$$M_{\mathrm{h}} + F_{\mathrm{h}}\frac{d}{2} \geq F_{\mathrm{PO}}a \quad \textbf{[1]}$$

where d and a represent the cell diameter and the contact radius. Goldasteh *et al.* reported M_{h} (*2*) as

$$M_h = \frac{2\pi\rho f_m d^3 u^2}{C_c} \quad \textbf{[2]}$$

where ρ, f_{m}, d, u and C_{c} represent the density of PBS (= 1.007×10^3 kg m^{-3}), the wall effect correction factor (= 0.943993 [3]), the cell diameter on the culture base (= 20 µm (*4*)), the shear velocity and Cunningham correction factor (≈ 1 (*2*)). On the other hand, F_{h} [2] is

$$F_h = \frac{3\Gamma\pi f\rho d^2 u^2}{2C_{\mathrm{c}}}(1 + 0.15Re^{0.678}) \quad \textbf{[3]}$$

where Γ, f, ρ, d, u, C_{c} and *Re,* respectively represent the linear profile in the viscous sublayer (= 1), the correction factor for wall effect (= 1.7009), the density of PBS (=10^3 kg m^{-3}), the cell diameter on the culture base (= 20 µm [4]), the shear velocity, Cunningham correction factor (≈ 1) and Reynolds number. With these expressions, we may now determine the left-hand side of eqn. **[1]** and produce the relationship

$$M_h \gg F_h\frac{d}{2}.$$

Additionally, F_{PO} of mammalian cells is 30–600 nN [5,6], with the range dependent upon the kind of cells and substrates, while *a* of CHO is 13.2 μm (calculated from Fig. 5B). From these calculations, the shear velocity required for cell detachment, *u*, is approximately 89–400 mm s^{-1}.

We also evaluated the pressure caused by acoustic radiation in our system. It has been determined that the pressure required to detach cells in past research is about 10–100 N m^{-2} [7], though in the presence of serum-free medium (SFM) the adhesion of the cells is expected to be reduced from these values. The measurement of the pressure amplitudes is difficult with even an extremely small pressure probe, due to the shallow PBS depth in the culture dish. Hence, we calculated the acoustic radiation pressure of traveling and standing waves by using King's equation[8]. The acoustic radiation pressure of a traveling wave, P_t, is

$$P_t = 2\pi\rho_P|A|^2(\kappa R_0)^6 \frac{9+2\left(1-\frac{\rho_C}{\rho_P}\right)^2}{9\left(2+\frac{\rho_C}{\rho_P}\right)^2} \quad \textbf{[4]}$$

and the acoustic radiation pressure of a standing wave, P_s, is

$$P_s = \frac{1}{3}\pi\rho_P|A|^2(\kappa R_0)^3 \frac{5-2\frac{\rho_C}{\rho_P}}{2+\frac{\rho_C}{\rho_P}} \sin(2kd) \quad \textbf{[5]}$$

where ρ_P, $|A|$, κ, R_0, ρ_C/ρ_P respectively represent the density of PBS ($\rho_P = 1.007 \times 10^3$ kg m^{-3}), the complex amplitude of the velocity potential of the incident wave ($|A| = 1.5 \times 10^{-6}$ m s^{-1}), its wavenumber ($\kappa = 2\pi f/c \approx 127$ m^{-1}), an estimated cell radius ($R_0 = 10$ μm) and the density ratio between cells and PBS ($\rho_C/\rho_P \approx 0.978$). From these equations, the maximum acoustic radiation pressure for traveling and standing waves is, respectively, $P_t = 6.74 \times 10^{-27}$ N m^{-2} and $P_s = 4.93 \times 10^{-18}$ N m^{-2}.

Since we used a frequency-swept wave in our experiments, the generated acoustic field is not constant, with a changing amplitude. Thus, an intermittent traveling wave is formed, and we calculated the pressure from knowledge of the acoustic impedances and amplitudes in a simple one-dimensional model. In the acoustic pressure model (Supplementary Fig. 3b), the pressure received by the cell was calculated from the difference in pressure between the top and bottom of the cell. The acoustic pressure in the vertical direction[9] is

$$P = -2\pi f \rho_l c_l A \sin(2\pi f t)$$

where P, f, ρ_l, c_l, A and t are represent the pressure, the freqency, the liquid density, the sound speed in the liquid, the amplitude and time. This produces a root mean squared magnitude for the acoustic pressure of

$$P_{rms} = \frac{2}{\sqrt{2}}\pi f Z_1 A \quad \textbf{[6]}$$

where Z_l is represent the acoustic impedance. The acoustic pressure developed upon a cell surrounded by PBS on the bottom of a cell, within the cell, and upon the top of a cell in our simple one-dimensional model are, respectively, P_{PB}, P_C, and P_{PT} as shown in Supplementary Fig. 3b. From eqn. **[4]**, P_{PB} is

$$P_{PB} = \sqrt{2}\pi f_{PB} Z_{PB} A_{PB} \quad \textbf{[7]}$$

where f_{PB}, Z_{PB} and A_{PB} are, respectively, the frequency of the acoustic wave (= 30.4 kHz), the acoustic impedance of PBS beneath the cells (= 1.565×10^6 kg m^{-2} s^{-1} [10]), and the amplitude of the acoustic vibration in the PBS beneath the cells (= 1.5 μm as shown in Supplementary Fig. 3c).

The relationship between the pressures and acoustic impedances[11] are given by

$$P_C = \frac{2Z_C}{Z_{PB} + Z_C} P_{PB}$$

and

$$P_{PT} = \frac{2Z_{PT}}{Z_C + Z_{PT}} P_C = \frac{2Z_{PT}}{Z_C + Z_{PT}} \cdot \frac{2Z_C}{Z_{PB} + Z_C} P_{PB} \quad \textbf{[8]}$$

where Z_C and Z_{PT} are, respectively, the acoustic impedances within the cells (= 1.6×10^6 kg m^{-2} s^{-1} [10]) and in the PBS atop the cells (= 1.565×10^6 kg m^{-2} s^{-1} [10]). From the calculation results above, the acoustic pressure produced in our system ($|P_{PT}$-$P_{PB}|$) is approximately 38.8 N m^{-2}, less than but comparable to the reported range of acoustic pressure caused by intermittent traveling wave necessary to detach cells. In the presence of the adhesion-reducing SFM, it may be sufficient.

We additionally evaluated the shear flow caused by sloshing from the use of sweep vibration in our system. In our simple order-of-magnitude model of the phenomena as shown in Supplementary Fig. 3c, the volume of fluid that crosses the boundary, right-to-left, V_C, is equivalent to the volume of fluid causes a height increase of the fluid interface, V_S, the observed *sloshing* in the video. Therefore, V_S is

$$V_S \approx r\Delta h \quad \textbf{[9]}$$

where r and Δh are respectively the fluid width (≈ 6 mm estimated from supplementary movie 1) and the increase in height of the fluid interface from sloshing (≈ 0.11 mm as measured by LDV), while V_C is

$$V_C \approx H\Delta L \quad \textbf{[10]}$$

where H and ΔL are respectively the height of PBS (= 2 mm) and the moving distance of PBS. From eqns. **[9,10]**, the horizontal fluid velocity on the dish, v, is

$$v = \frac{\Delta L}{\Delta t} = \frac{r}{H} f_S \Delta h \quad \textbf{[11]}$$

where Δt and f_s are respectively the duration time and the frequency of sloshing (≈ 45 Hz calculated from movie S1, which corresponds to the frequency of the sweep signal used in the experiments). From the calculation results above, the flow velocity generated by sloshing of sweep vibration in our system (v) is approximately 14.9 mm s^{-1}, less than the shear velocity required for cell detachment, but significantly more than the acoustic streaming. Further, using continuous vibration input instead significantly reduces the cell detachment, and consequently it can be concluded that the sloshing phenomena induced by the frequency sweeping of the input signal is an important factor in the cell detachment alongside the acoustic pressure caused by the intermittent traveling wave and the adhesion-reducing SFM.

Detachment data of multiple cell types.

For the usefulness of the detachment method using acoustic pressure and sloshing, we demonstrated the ability to detach cells for three different types of cell lines: a mouse myoblast, C2C12, a human mesenchymal stem cell (MSC), UE7T and human cervical cancer cells, HeLa. These cells were detached when R_{id} was 0.875 and detachment conditions were the same as CHO cells. As shown in Supplementary Fig. 6a, no significant difference was found in the number of cells detached by the proposed method and trypsinization in all cell types. Additionally, the numbers of cells detached by the proposed method were statistically larger than those by trypsinization after 48 h (Supplementary Fig. 6b).

Supplementary References

1. Soltani, M. & Ahmadi, G., Particle removal mechanisms under substrate acceleration. *J Adhes* **44**, 161–175 (1994).

2. Goldasteh, I., Shmadi, G. & Ferro, A. R., Monte Carlo simulation of micron size spherical particle removal and resuspension from substrate under fluid flows. *J Aerosol Sci* **66**, 62–71 (2013).

3. O'neill, M. E., A sphere in contact with a plane wall in a slow linear shear flow, *Chem Eng Sci* **23**, 1293–1298 (1968).

4. Yum, K., Wang, N., & Yu, M-F., Nanoneedle: A multifunctional tool for biological studies in living cells, *Nanoscale* **2**, 363–372 (2010).

5. Potthoff, E., *et al*., Rapid and serial quantification of adhesion forces of yeast and mammalian cells, *PLoS ONE* **7**, e52712 (2012).

6. Sagvolden, G., Giaever, I., Pettersen, O. E. & Feder, J., Cell adhesion force microscopy, *Proc Natl Acad Sci USA* **96**, 471–476 (1999).

7. Christophis, C., Grunzeab, M., & Rosenhahn, A., Quantification of the adhesion strength of fibroblast cells on ethylene glycol terminated self-assembled monolayers by a microfluidic shear force assay. *Phys Chem Chem Phys* **12**, 4498–4504 (2010).

8. Louis, V. & King, F. R. S., On the acoustic radiation pressure on spheres, *Proc. Royal Soc. Lond* **15**, 212–240 (1934).

9. Kim, K-H., Chahine, G., Franc, J-P. & Karimi, A., *Advanced experimental and numerical techniques for cavitation erosion prediction* (Springer, Netherlands, 2014).

10. Fadhel, M. N., Berndl, E. S., Strohm, E. M. & Kolios,. M. C., High-frequency acoustic impedance imaging of cancer cells, *Ultrasound in Med & Biol* **41**, 2700–2713 (2015).

11. Kinsler, L. E., Frey, A. R., Coppens, A. B. & Sanders, J. V., *Fundamentals of acoustics* (Wiley Yew York 2009).
