# Supplementary material for: Enzyme-free release of adhered cells from standard culture dishes using intermittent ultrasonic traveling waves
Source: Commun Biol. 2019 Oct 29;2:393. doi: 10.1038/s42003-019-0638-5 (PMC6820801; doi:10.1038/s42003-019-0638-5)
Supplement: Supplementary file 2 — Description of Additional Supplementary File [file 42003_2019_638_MOESM2_ESM.docx]

**Description of Additional Supplementary Files**

**I) File Name: Supplementary Movie 1**

Description: Fluid surface motion in the culture dish with the sweep vibration. The movie was recorded from above at 30° from the vertical in order to visualize the motion via specular reflection of light from the fluid interface. The movies captured by high-speed camera was 1000 fps, and the provided movie is encoded to play at 15 fps.

**II) File Name: Supplementary Data 1**

The .xlsx file contains 15 sheets related to figures 2–7, from the main text. The data of a given figure is always preceded by the figure identity in bold (ex. **Fig. 1b**).
