# Supplementary material for: Enzyme-free release of adhered cells from standard culture dishes using intermittent ultrasonic traveling waves
Source: Commun Biol. 2019 Oct 29;2:393. doi: 10.1038/s42003-019-0638-5 (PMC6820801; doi:10.1038/s42003-019-0638-5)
Supplement: Supplementary file 5 — Reporting Summary [file 42003_2019_638_MOESM5_ESM.pdf]

nature research

Corresponding author(s): Yuta Kurashina, Kenjiro Takemura

Last updated by author(s): Sep 14, 2019

Reporting Summary

Nature Research wishes to improve the reproducibility of the work that we publish. This form provides structure for consistency and transparency in reporting. For further information on Nature Research policies, see Authors & Referees and the Editorial Policy Checklist.

Statistics

For all statistical analyses, confirm that the following items are present in the figure legend, table legend, main text, or Methods section.

n/a	Confirmed	
☐	☒	The exact sample size (*n*) for each experimental group/condition, given as a discrete number and unit of measurement
☐	☒	A statement on whether measurements were taken from distinct samples or whether the same sample was measured repeatedly
☐	☒	The statistical test(s) used AND whether they are one- or two-sided<br>*Only common tests should be described solely by name; describe more complex techniques in the Methods section.*
☐	☒	A description of all covariates tested
☒	☐	A description of any assumptions or corrections, such as tests of normality and adjustment for multiple comparisons
☐	☒	A full description of the statistical parameters including central tendency (e.g. means) or other basic estimates (e.g. regression coefficient) AND variation (e.g. standard deviation) or associated estimates of uncertainty (e.g. confidence intervals)
☐	☒	For null hypothesis testing, the test statistic (e.g. *F*, *t*, *r*) with confidence intervals, effect sizes, degrees of freedom and *P* value noted<br>*Give P values as exact values whenever suitable.*
☒	☐	For Bayesian analysis, information on the choice of priors and Markov chain Monte Carlo settings
☒	☐	For hierarchical and complex designs, identification of the appropriate level for tests and full reporting of outcomes
☒	☐	Estimates of effect sizes (e.g. Cohen's *d*, Pearson's *r*), indicating how they were calculated

Our web collection on statistics for biologists contains articles on many of the points above.

Software and code

Policy information about availability of computer code

Data collection	N/A
Data analysis	N/A

For manuscripts utilizing custom algorithms or software that are central to the research but not yet described in published literature, software must be made available to editors/reviewers. We strongly encourage code deposition in a community repository (e.g. GitHub). See the Nature Research guidelines for submitting code & software for further information.

Data

Policy information about availability of data

All manuscripts must include a data availability statement. This statement should provide the following information, where applicable:

- Accession codes, unique identifiers, or web links for publicly available datasets
- A list of figures that have associated raw data
- A description of any restrictions on data availability

All data supporting this study are presented here (or as supplementary information).

Field-specific reporting

Please select the one below that is the best fit for your research. If you are not sure, read the appropriate sections before making your selection.

☒ Life sciences ☐ Behavioural & social sciences ☐ Ecological, evolutionary & environmental sciences

For a reference copy of the document with all sections, see nature.com/documents/nr-reporting-summary-flat.pdf

Life sciences study design

All studies must disclose on these points even when the disclosure is negative.

Sample size	Sample size was determined based on extensive previous experience in the field; no statistical methods were used to predetermine this.
Data exclusions	no data were excluded.
Replication	All attempts for reproducibility were successful.
Randomization	All allocations were random.
Blinding	All cell experiments were blinded.

Reporting for specific materials, systems and methods

We require information from authors about some types of materials, experimental systems and methods used in many studies. Here, indicate whether each material, system or method listed is relevant to your study. If you are not sure if a list item applies to your research, read the appropriate section before selecting a response.

Materials & experimental systems

n/a	Involved in the study	
☐	☒	Antibodies
☐	☒	Eukaryotic cell lines
☒	☐	Palaeontology
☒	☐	Animals and other organisms
☒	☐	Human research participants
☒	☐	Clinical data

Methods

n/a	Involved in the study	
☒	☐	ChIP-seq
☒	☐	Flow cytometry
☒	☐	MRI-based neuroimaging

Antibodies

Antibodies used	Primary antibodies: Rabbit anti-uman LIFR polyclonal antibody (22779-1-AP; Proteintech, Rosemont, IL, USA), a 1,000-fold dilution of rabbit anti-human Integrin α5 (D7B7G) monoclonal antibody (#98204; Cell Signaling Technology, Boston, MA, USA), and a 1,000-fold dilution of rabbit anti-mouse β-actin polyclonal antibody (#4967; Cell Signaling Technology).<br>Secondary antibody: Horseradish peroxidase (HRP)-labeled secondary antibody (1:10,000 dilution, HRP-conjugated anti-rabbit IgG; Perkin-Elmer, Waltham, MA, USA)
Validation	As described manufacturer's website.

Eukaryotic cell lines

Policy information about cell lines

Cell line source(s)	Chinese hamster ovary cells: CHO-K1 line and mouse myoblast (C2C12): RCB0987 from Riken Bio Resource. Human mesenchymal stem cells: UE7T-13 line and HeLa cells: JCRB9004 line from JCRB.
Authentication	Based on source authentication
Mycoplasma contamination	JCRB and Riken cell bank tested for mycoJCRB and Riken tested fluorescence staining method or PCR method for mycoplasma contamination before provide.plasma contamination before provide.
Commonly misidentified lines (See ICLAC register)	N/A
